# Supplementary material for: Monocyte MRI Relaxation Rates Are Regulated by Extracellular Iron and Hepcidin
Source: Int J Mol Sci. 2023 Feb 17;24(4):4036. doi: 10.3390/ijms24044036 (PMC9962677; doi:10.3390/ijms24044036)
Supplement: Supplementary file 1 [file ijms-24-04036-s001.zip › ijms-2165229-supplementary.pdf]

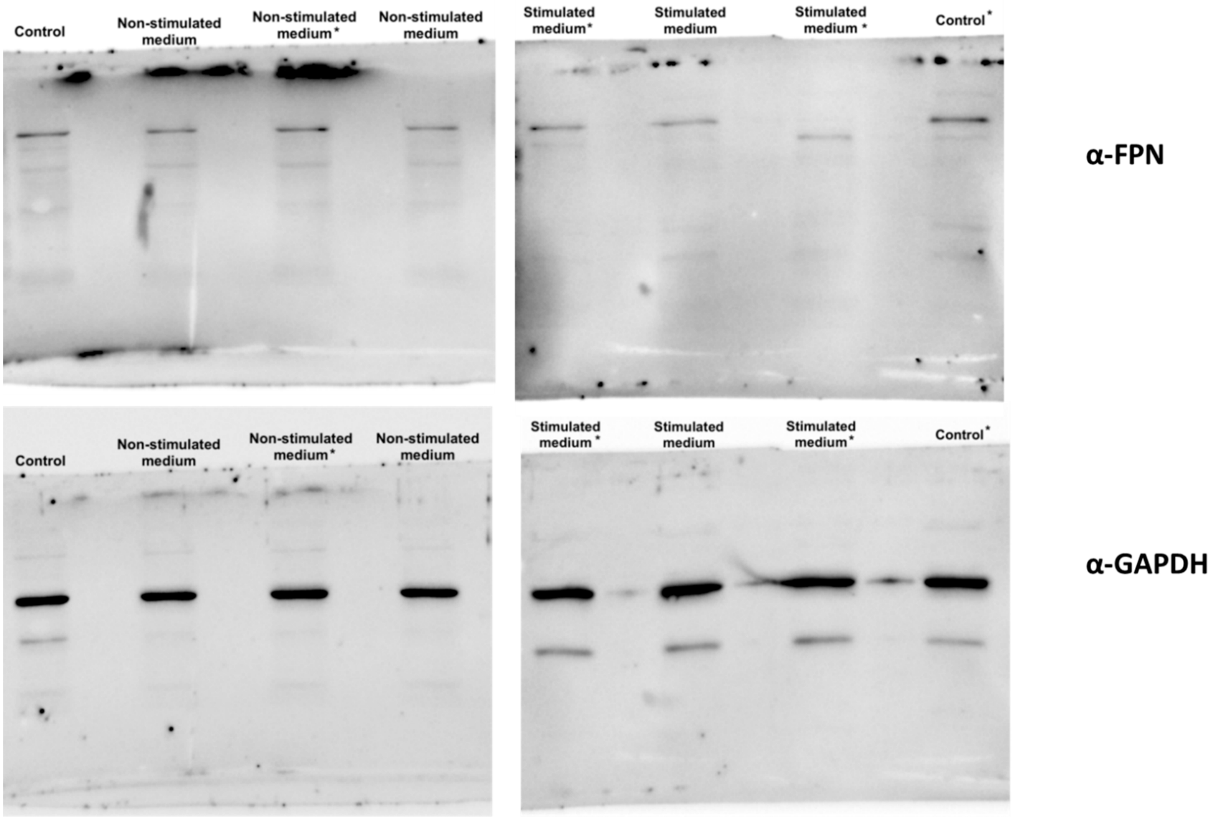

Figure S1. Regulation of ferroportin levels in monocytes by secreted hepcidin activity. THP-1 cells were cultured in the absence (−Fe, control medium) or presence of P19 cell-conditioned medium, either non-stimulated or stimulated to secrete hepcidin. Representative blots identify protein levels using antibodies to ferroportin (α-FPN, upper panels) and glyceraldehyde-3-phosphate dehydrogenase (α-GAPDH, lower panels). Representative samples included in Figure 2A are denoted with an asterisk (*).
